# Neural Correlates of Consciousness Meet the Theory of Identity

**DOI:** 10.3389/fpsyg.2018.01269

**Published:** 2018-07-24

**Authors:** Michal Polák, Tomáš Marvan

**Affiliations:** ^1^Department of Philosophy, Faculty of Philosophy and Arts, University of West Bohemia, Pilsen, Czechia; ^2^The Czech Academy of Sciences, Institute of Philosophy, Prague, Czechia

**Keywords:** neural correlates of consciousness (NCCs), type identity theory, phenomenal states, type-token, non-causal account of NCC

## Abstract

One of the greatest challenges of consciousness research is to understand the relationship between consciousness and its implementing substrate. Current research into the neural correlates of consciousness regards the biological brain as being this substrate, but largely fails to clarify the nature of the brain-consciousness connection. A popular approach within this research is to construe brain-consciousness correlations in causal terms: the neural correlates of consciousness are the causes of states of consciousness. After introducing the notion of the neural correlate of consciousness, we argue (see Against Causal Accounts of NCCs) that this causal strategy is misguided. It implicitly involves an undesirable dualism of matter and mind and should thus be avoided. A non-causal account of the brain-mind correlations is to be preferred. We favor the theory of the identity of mind and brain, according to which states of phenomenal consciousness are identical with their neural correlates. Research into the neural correlates of consciousness and the theory of identity (in the philosophy of mind) are two major research paradigms that hitherto have had very little mutual contact. We aim to demonstrate that they can enrich each other. This is the task of the third part of the paper in which we show that the identity theory must work with a suitably defined concept of *type*. Surprisingly, neither philosophers nor neuroscientists have taken much care in defining this central concept; more often than not, the term is used only implicitly and vaguely. We attempt to open a debate on this subject and remedy this unhappy state of affairs, proposing a tentative hierarchical classification of phenomenal and neurophysiological types, spanning multiple levels of varying degrees of generality. The fourth part of the paper compares the theory of identity with other prominent conceptions of the mind-body connection. We conclude by stressing that scientists working on consciousness should engage more with metaphysical issues concerning the relation of brain processes and states of consciousness. Without this, the ultimate goals of consciousness research can hardly be fulfilled.

## Introduction

Tracking the correlations between brain processes and states of phenomenal consciousness, such as feelings of pain, seeings of blue, hearings of trumpet sounds, is the basic method of scientific consciousness research.^1^ Searching for such correlations with the help of modern brain imaging techniques has produced, since its inception in the 1990s, a body of remarkable results and a number of competing hypotheses regarding the neural correlates of conscious experience within different sensory modalities (Frith et al., 1999; Gallace and Spence, 2008; Ionta et al., 2011; Fleming and Dolan, 2012; Merrick et al., 2014; Koch et al., 2016). Although as yet there is no consensus on how to interpret the multifarious data, the search for the neural correlates of consciousness in the brain is clearly the first major step toward a robust empirical theory of consciousness.

The notion of correlation leaves unresolved the precise nature of the relationship between subjective phenomenal states and neurophysiological processes. Francis Crick, one of the initiators of the search for the neural correlates of consciousness, emphasized that he used the word “correlate” as an ontologically *neutral* term. Such and such neural activity is detected, and it is accompanied by a certain subjective experience (Crick and Koch, 1990). As a first step this is acceptable but from the explanatory point of view, this cannot be the last word. The relationships between the two co-relata need be further clarified if we are to understand why the correlation exists in the first place. Most probably, the actual connection between mind and body is tighter than just a brute correlation of their states. Thus what kind of a metaphysical relation holds between brain processes and subjectively experienced states, such that they reliably co-occur?

The theory of the identity of mind and body, formulated more than half a century ago by Place (1956) and Smart (1959), gives a straightforward answer: according to this theory, states of consciousness *are* brain processes and because of that, they systematically correlate with these processes. Thus, for example, a feeling of pain in my hip is identical to an activation of some area (or areas) in my brain, and this explains their regular correlation. In Smart’s version, the theory was a materialist attempt to discredit dualist accounts of mind-brain correlation, according to which the correlation is based upon a regular interaction between corporeal and incorporeal processes. The theory was formulated before the heyday of the modern science of consciousness and the brain imaging technology it employs. However, we believe that even today the theory can be used to sufficiently clarify the relation between brain and consciousness. It is a simple, elegant tool for interpreting the results of neuroscientists’ lab-based mind-brain correlation measurements. There are other prominent metaphysical accounts of the brain/consciousness relationship. In this paper we shall show the advantages the theory of identity holds over those alternative accounts. Our second goal is to propose a working definition of a phenomenal and a neurophysiological type. Measurements of the neural correlates of consciousness focus on particular instances of mind-brain correlations, the relata of which are individual phenomenal and neurophysiological “tokens.” However, the obvious goal of this enterprise is to generalize the token measurements into systematic type–type mind-brain correlations. Still, the notion of a phenomenal and a neurophysiological type remains far from clear.

Before delving into these questions, a word is needed about the very notion of the neural correlate of consciousness (or NCC, for short). In a widely cited definition, Chalmers (2000, p. 31) delimits the NCC as a *minimal and sufficient neural system N* whose activation leads to a conscious percept. This is basically correct, but needs to be somewhat modified. We will follow Fink (2016) in taking the NCC to be not a neural system itself, but an *activation* of this system. Fink’s re-definition (“NCC 2.0”) is not only more precise than Chalmers’ but is congenial to our way of proceeding because it works with the distinction between tokens and types. In full, his definition reads:

“NCC2.0: An NCC2.0 of a phenomenal *type P* is a *type* of neural event or process *N* such that there is a mapping, where (i) each neural token *n_i_* of *N* is minimally sufficient for a phenomenal token *p_i_* of *P*, and (ii) where all and only neural tokens of *N* instantiate a feature-bundle 𝔽, such that 𝔽 is a (naturally) *necessary* condition for being an NCC of *P*.” (Fink, 2016, p. 9)^2^

This definition is compatible with the sort of the theory of identity which identifies mental and neural phenomena at the level of types – and it is upon this version of the theory that we shall focus. We have two things to add to Fink’s NCC2.0 definition. First, it requires “natural necessity” (the necessity of a natural law) at the level of types. We do not want to commit to such a strong requirement, for reasons which will emerge at the end of section “A Plea for Identity Theory in Consciousness Research”. *Sufficiency* at the type-level should be enough. Secondly, the neural “feature-bundle,” according to Fink, is a general mechanism of consciousness, such as, for instance, recurrent neural activity. This makes little sense even in the light of Fink’s own goals. The neural type-mechanisms need to be content-specific; they need to be able to articulate distinct phenomenal states. Thus, we propose to modify Fink’s definition in the following manner:

NCC2.1: An NCC2.1 of a phenomenal *type P* is a *type* of neural event or process *N* such that there is a mapping, where (i) each neural token *n_i_* of *N* is minimally sufficient for a phenomenal token *p_i_* of *P*, and (ii) where all and only neural tokens of *N* instantiate **a neural mechanism M**, such that **M** is a ***sufficient*** condition for being an NCC of *P*.

Our aim in this paper is not to adjudicate between various proposals as to what constitutes an NCC. Whatever is the right NCC of the given type of a conscious mental state, it is identical with this state, we claim. What we shall suggest should be easy to adjust for all relevant NCC theories discussed in the contemporary literature.^3^

## Against Causal Accounts of NCCs

**FIGURE 1:**
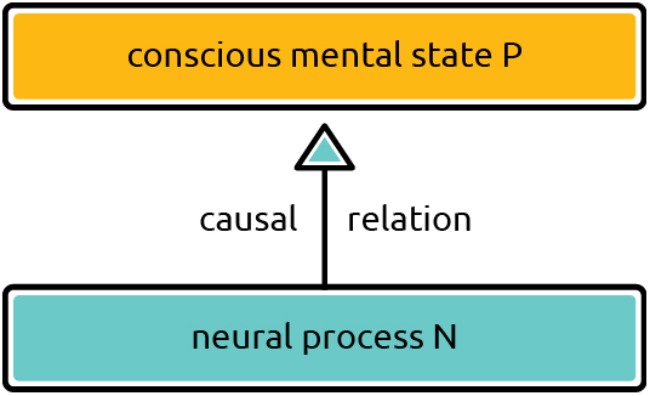
Causal account of NCC. Phenomenal conscious state P is caused by neural process N. A causal explanation is often appealed to in empirical accounts of mind-brain relations.

Most contemporary attempts at accounting for brain-consciousness correlations are materialist. States of consciousness are not states of some mysterious immaterial substance; they are in some way anchored in detectable and measurable brain states. However, even within the materialist framework the correlation can be explained in various ways. Regular mind-brain correlations form an evidentiary basis which can be used in support of different metaphysical accounts. The most straightforward approach is to try to explain the correlations in causal terms: NCCs are the causes of the states of consciousness. To “go from correlation to causation” is a move typical of the sciences and it might seem intuitively appealing to treat brain states as the causal sources of states of consciousness. No wonder, then, that we find advocates of the causal interpretation of NCCs. Neisser (2012); Sergent and Dehaene (2012), and Wessel (2012) put forward such causal accounts. Crick and Koch (2003), the founders of the field of modern consciousness studies, also want to “explain NCC in causal terms.” Still, we believe that this explanatory strategy is deeply problematic. It is far less plausible than the identity account of mind-brain correlation. To see why, consider the picture put forward by the causal accounts. A neurophysiological process *causes* a phenomenal state of consciousness; therefore, it is *different* from that state, because causes and effects are always distinct. **Figure 1** shows a sketchy diagram of the view that a neural process N causes a conscious mental state P.

However, materialist principles dictate that every conscious state must be implemented materially, i.e., by some brain state(s). Since the conscious state is different from the neurophysiological processes that are causing it, it must, on pain of psycho-physical dualism (cf. Fell et al., 2004), be implemented by a material process distinct from its neural cause. Thus we end up with *two* material processes involved in the production of the conscious mental state, not one. The first material brain process would be, according to the causal approach, the cause of a conscious state. The second neural process then would be the implementation of the phenomenal conscious state P, though it would not be its cause. Without this second material process the conscious state would not have a place in a materialist universe.

Which of these two neural processes is to be considered the *real* NCC? Clearly it is the *second* kind. The first kind of process undoubtedly regularly correlates with occurrences of the state of consciousness at hand, because it is its regular cause and regular causes do correlate with their regular effects. Let us call this kind of brain process the Causal Neural Correlate of Consciousness (CNCC). Yet the CNCC is not what the cognitive neuroscientists empirically investigating consciousness are primarily searching for. They are searching for the brain processes of the second kind. Take as an example the firings of C-fibers, beloved by philosophers of mind (see, e.g., Kripke, 1980). These firings are, in fact, the CNCCs of some kinds of pain sensations, because they are the causal antecedents of these sensations. Being a subset of sensory neurons projecting to the spinal cord, C-fibers are not even a part of the brain areas where these kinds of pain sensations arise.^4^ They are not its *mechanism* – that is, the activation of what is called by Swanson (2012, pp. 252–253) the central nociceptive system in the brain, which probably comprises parts of prefrontal cortex and the rostral half of the insular cortex.

**FIGURE 2:**
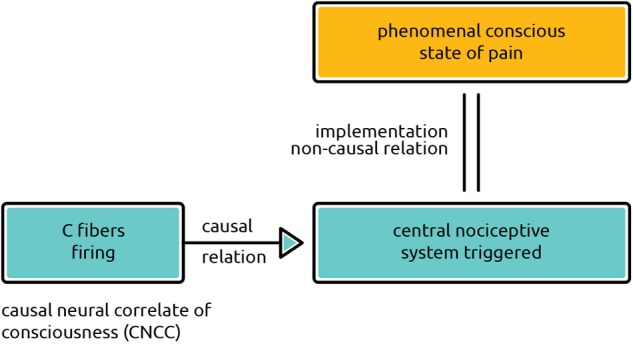
Non-causal account of NCC. A phenomenal conscious state of pain is not caused, but non-causally implemented by its true neural correlate. The *causal* neural correlate of consciousness (CNCC; firing C-fibers in the diagram) precedes in time the activation of the true NCC (in the diagram, the true NCC is the activated central nociceptive system in the prefrontal-insular cortex).

Causal neural correlates of consciousness precede in time the real or “true” (Sandberg et al., 2016) NCCs (see **Figure 2**). They are sometimes labeled the “upstream” correlates of NCC, or *NCCpre*. Apart from them, one can also distinguish the *NCCco* or “downstream” correlates of consciousness. Both the neural antecedents and consequents are confounders of the true NCCs. Thus the true NCC is simply what remains after we cut off the various confounders; and, we suggest, the true NCC is what is captured by the mind-brain theory of identity. The NCC, we might say, is an *identity-correlate* of a conscious phenomenal state.

**FIGURE 3:**
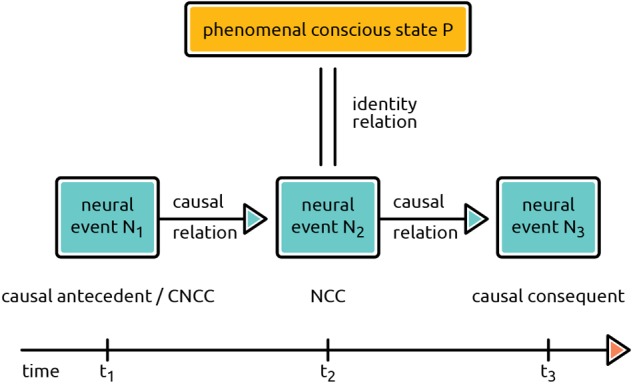
Horizontal and vertical NCCs. The diagram distinguishes between horizontal and vertical relations and isolates the real NCC – neural event N_2_ in the diagram. It is this event N_2_ that is to be identified with phenomenal conscious state P. The diagram is based on Hohwy and Bayne (2015, p. 156); modified.

The preceding train of thought is, we believe, sufficient to demonstrate that the relation between states of phenomenal consciousness and brain states correlating with them cannot be causal, provided that a materialistic account of consciousness is correct. Empirical studies of consciousness, however, do not always distinguish between the causal and non-causal relations of phenomenal and neural states. For instance, Gallotto et al. (2017), after distinguishing “neural substrates” (i.e., NCCs) from both the neural prerequisites and neural consequences of a conscious experience, proceed to affirm that the neural substrates of experience “are directly *causing*, or are *identical with*, the phenomenal conscious experience” (p. 10; italics added). However, one *cannot* have both at the same time. These relations are fundamentally different. To avoid this confusion, it is profitable to distinguish between *horizontal* and *vertical* NCCs (Hohwy and Bayne, 2015). Horizontal causal relations obtain between the proper NCC and its confounds, i.e., its causal antecedents and consequents. The notion of a vertical NCC concerns the very relation between a neural event and a phenomenal conscious state. (See **Figure 3**).

Horizontally, neural events N_1_, N_2_, and N_3_ are related causally. The true NCC (N_2_) is thus causally involved in a horizontal structure of neural events. On the other hand, only N_2_ is involved in the vertical relation between the neural event and the phenomenal conscious state P.^5^

This paper is about the vertical relations between NCCs and their corresponding states of consciousness. These relations cannot be causal because attempts to treat vertical relations in a causal manner lead to the confusions described above. To accept only non-causal vertical relations means that not only the simple and straightforward causality, but also the more nuanced causal relations such as *emergence*^6^ cannot be admitted as vertical brain-mind relations. Identity, on the other hand, is not a causal relation, and thus can, in principle, serve as an appropriate candidate for the relation between conscious states and their NCCs. In the following section we shall consider how a theory of identity for the mind-brain relation should look. This will inevitably require an inquiry into the notion of a mental and a neurophysiological type.

## The Science of Consciousness Needs a Notion of Type

Scientists searching for NCCs are measuring particular instances of mind-brain correlations, but this is hardly the end-point of their inquiry. They aim to generalize their findings. They want to know not only the neural correlate of a particular phenomenal token, say, the sensation of a sour taste. To put forward an infinite disjunction of particular phenomenal-neural correlations does not sound an attractive research project! The hope is that by accumulating a sufficient number of particular NCCs, the scientists will be in a position to generalize the data in some meaningful way. They will proceed by typing the phenomenal states into basic kinds and will try to assign to them broadly typed neurophysiological processes, carefully isolated during the experiments. This process needs to be repeatable both intrasubjectively and intersubjectively; hence it cannot stop at the level of individual tokens but must involve types. As Fink (2016, p. 3) put it, it is because science aims at generality that “we aim at *types*-NCCs.” This process can begin very crudely, but a rough and ready form of phenomenal and neural type-taxonomy is at least a prerequisite of any empirical science of consciousness. Even if the consciousness scientist sets herself relatively unambitious goals – say, she just wants to compile a list of some law-like bi-directional correlations between phenomenal and neural states – she will need to rely on some notion of phenomenal and neural type, for it will be types of states and processes that eventually have to appear in this list of systematic correlations. A general form of such bi-directional correlations will be, *Whenever a phenomenal state of type A is present, a neural process of type B is present* (and vice versa).

A solution to the vertical mind-body-relation problem, though, need not inevitably appeal to types. The so-called *weak* (or “token-token”) theory of identity postulates identity only at the level of individual tokens of mental and neural events. However, as the advocates of the weak theory of identity (such as Davidson, 1970/2012; Fodor, 1974) admit, the token-token theory of identity does not permit systematization into law-like psycho-physical generalizations: individual instances of mental and neural events cannot be regimented into correlated type-sets.^7^ The stronger, *type-type* theory of identity identifies types of conscious mental states with types of brain states and thus allows for systematic psycho-physical type-generalizations. Various forms of this stronger identity theory have been offered over the years (Place, 1956, 1988; Feigl, 1958; Smart, 1959; Lewis, 1966; Armstrong, 1968; Bechtel and Mundale, 1999; Polger, 2011; Polger and Shapiro, 2016; see also Gozzano and Hill, 2012). Debates within the philosophy of mind are quite extensive as regards the facets of the identity theory, its pros and cons (see Polger, 2009, for an overview), but very little attention has been given to the pivotal question of what the neurophysiological and phenomenal kinds are. Most theorists participating in these debates use the notions of phenomenal and neurophysiological types only intuitively, without giving any explicit principles of individuation. The same can be said of the empirical scientists of consciousness.

This absence of a common understanding of what constitutes a type is striking, given the centrality of the notion of *type* within both philosophy and neuroscience. Is pain a type of phenomenal state and the pain of a bee sting, the pain of a papercut etc. its instances? Or is the pain of a bee sting the type, and each individual instance of bee sting pain its token? The literature does not give any (decisive) answers to these questions. Usually, within the philosophy of mind, one or two examples of a phenomenal type are offered (pain and color perception are the most often used), and the assumption is that the reader will somehow get the whole idea. The brain side of things is hardly less nebulous: how we are to understand the notion of type in the neurophysiological domain is usually left underspecified. We shall try to remedy this by providing a tentative categorization of both kinds of types.

### Phenomenal Types

As Chalmers suggested, phenomenal types are to be typed by their phenomenal features alone (Chalmers, 1996, 359 n. 2). The typing of phenomenal types is thus straightforward, because kinds of subjective experience are clearly distinct: think of visual and auditory sensations and the vivid differences between them. The criteria for distinguishing phenomenal types are subjective, because these mental states are not publicly available. However, subjective judgments inevitably constitute the data of scientific practice (Jack and Shallice, 2001; Piccinini, 2001; Chaminade and Decety, 2002; Jack and Roepstorff, 2003; Price and Aydede, 2005; Overgaard, 2006; Block, 2008). This notion of type is what we have in mind when discussing the phenomenal types below.

We propose a hierarchical classification of phenomenal and neurophysiological types, spanning multiple levels of varying degrees of generality. We begin with the phenomenal types. At the top of the hierarchy are the most general kinds of types of conscious experience. At the bottom in the nomenclature we put “minimal types.” Between the general and the minimal types are all the in-between kinds of types, which form the focus both of philosophers of mind and cognitive neuroscientists in their research.

The highest category within the hierarchy comprises the most general types of sensations and feelings: pain, visual perception, taste, auditory perception, depression, etc. Take pain as an example. The International Association for the Study of Pain (2018) defines pain as “an unpleasant sensory and emotional experience.”^8^ The definition mirrors the intuition about what the most general kind of a phenomenal type should be like. In the case of pain it has to be something that hurts and is felt to be unpleasant. Defined in this general way, pain includes an extensive number of tokens of pain. These may be, for example, sharp pain, throbbing pain, stabbing pain caused by a needle, etc. as they all are tokens of pain. The most general phenomenal types are characterized by the highest variability among their tokens. The more we proceed down the hierarchy, the less variability is present among tokens within a type.

Before we turn to the in-between types, the minimal type should be characterized. A type is minimal when a subject cannot or can barely distinguish any phenomenal difference between at least two different subjective experiences. Types are minimal in this sense, regardless of whether there is a difference in the corresponding external stimuli or not. For example, if someone pricks me with a needle and later on with a knife and in each case I feel no difference in my phenomenal experience, the phenomenal type is of a minimal kind. The same applies when the two indistinguishable or nearly-indistinguishable experiences are caused by the same external stimulus. Minimal type is thus defined as having a minimal or no variability in the phenomenal character of its tokens.

**FIGURE 4:**
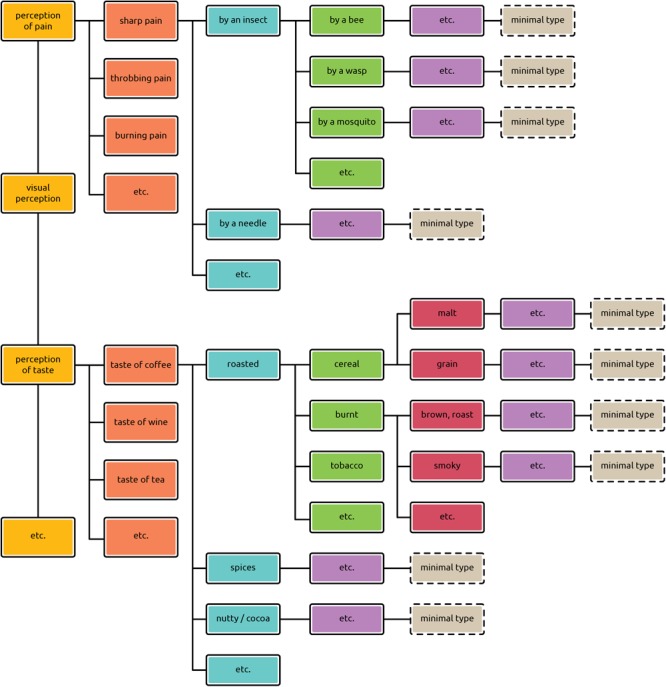
Hierarchical classification of phenomenal types. The hierarchy shows the most general types (perception of pain, visual perception, perception of taste, etc.) and, proceeding down, the less general types. (The subdivisions are sketched only for selected types.) The lower the type in the hierarchy, the lesser the variation between its instances. Minimal types manifest very little or no variability among their tokens.

The in-between categories of types include tokens of all perceptual modalities: seeing blue, hearing middle C, smelling a rose, tasting a Camembert, fear upon seeing a spider, etc. Each of these sub-categories can be further divided into finer-grained ones, and these, in turn, can be divided into even subtler ones; and so on. Thus the feeling of pain may be divided into more specific types such as the feeling of sharp pain, the feeling of throbbing pain, the feeling of burning pain, the feeling of cramping pain, the feeling of shooting pain, etc. Analogically, each of these types may be further split into finer-grained types such as the feeling of sharp pain caused by a needle, the feeling of sharp pain caused by an insect, etc., which again may be divided into even finer categories (see **Figure 4**). Each of these types is constituted by the individual instances of painful feelings which fall under it.

To repeat, phenomenal types are classified exclusively by their phenomenal appearance. For instance, coffee tasters do not need to have any knowledge of how the particular token of coffee they are tasting has been made; they just taste it and describe it according to its phenomenal properties. Knowledge of the causes of the sensations does not enter into the classification of the kinds of types subsumed in **Figure 4** under the type *taste of coffee*, or of any other of the phenomenological sub-types mentioned. Even if the sub-types are labeled using the vocabulary of the causes of sensations, such as in the case of kinds of pain, the reference to causes serves simply to verbally differentiate between various painful feelings.

Not only empirically oriented philosophers of mind but also those scientists conducting consciousness research should be clear about where in the hierarchy the phenomenal types they are investigating are located. For instance, *pain*, one of the most commonly mentioned phenomenal types, is at a different level of generality from *smelling an orange* (Keaton, 2015, p. 396), *fudgefeel* (Tye, 1995, p. 54) and *seeing a face* (Lumer et al., 1998). Clarity regarding the target level of the phenomenal type is important because the corresponding neural types have to be located at the same level of generality.

### Neural Types

The theory of identity postulates a correspondence between phenomenal and neurophysiological types at each level of generality. It is not obvious, though, which kinds of neural types match the phenomenal ones. Phenomenal types are typed by their phenomenal properties, whereas neural types are sorted by means of entirely distinct sets of characteristics. Usually, the existence of some neural type of mechanism is suggested, such as the infamous firing of C-fibers, and although this particular example is empirically wrong, it indicates a general approach to fixing neural types. The idea is to isolate the activated mechanism in the brain directly responsible for particular type of a conscious phenomenal experience. This can be done by repeating the measurements of brain states correlating with the tokens of the given phenomenal type. This is what the NCC research aims to do: to uncover the neural types by means of finding the same (or similar) pattern of neural activation across many tokens of the same (or relevantly similar) phenomenal experience.

Putting it this way gives pride of place to the practices of contemporary cognitive neuroscience. It locates neural types at the level of activated brain structures and neural populations. Cognitive neuroscientists studying, for example, the neural correlates of visual experience know that they have to devote most attention to the neural processes arising in areas such as V1, V2, V3, V4, MT and in some other parts of the temporo-parietal cortex. The fact that these areas are preferred over many other cortical sites is rooted in knowledge acquired over decades of empirical inquiry. The research has aimed to isolate the circumscribed anatomical areas responsible for specific functions; cf. Broca’s studies on aphatic patients (Sobel, 2001). We believe that respecting the practices of cognitive neuroscience is a pragmatically sensible approach, although we do not want to disqualify other possible approaches to neural typing.^9^

The practices of cognitive neuroscience place NCCs and thus the neural types firmly in the cerebral cortex. For example, it is a well-founded supposition that all NCCs are located within temporo-parieto-occipital cortical areas.^10^ Contributions from subcortical structures such as the brainstem, reticular formation, hypothalamus and thalamus are necessary to sustain conscious states. However, these contributions are routinely ignored in the NCC research and there are even more systematical reasons for disregarding them: clinical and experimental findings show cases of extensive activity in the cerebral cortex even when the subcortical neuromodulatory systems, such as the reticular activation system, are unplugged. For example, during REM sleep people dream consciously although the cerebral cortex is disconnected from subcortical structures (Koch et al., 2016, p. 310).

Does the restriction to the cortex imply that a precise localization within cortex structures is part and parcel of neural typing? That could well be doubted. On the one hand, the localization thesis is to a large extent respected in neurosciences. Neuroscientists do not expect that for every individual case of the occurrence of a phenomenal state, its neural correlate can be found everywhere in the brain. The current debate is mostly about the mechanisms involved in visual perception and the functional specialization of individual visual areas or their sub-parts. Researchers do not expect to find correlates of visual consciousness in the extrastriate cortex in one experiment, in the anterior cingulate cortex in the following experiment and in dlPFC in the next experiment. They expect a degree of systematicity in how the NCCs of various types of phenomenal states are localized. Without this presumption, much of cognitive neuroscience would simply fall apart.

On the other hand, phenomena such as neural plasticity suggest that the localization of neural types is not absolute. Some phenomenal types may be implemented by different parts of the brain. The neural type, then, is the neurophysiological process, the mechanism, whatever it may be, sufficient for having a conscious phenomenal state. Localizing the processes in the brain is heuristically important; it helps us to differentiate brain areas due to their functional properties so that it is subsequently possible to concentrate on the neural pattern-properties themselves. However, isolating the neural mechanisms of consciousness is *the* project which constitutes the search for neural types. These mechanisms can, due to lesions, for instance, neuroplastically shift their locations somewhat, without ceasing to be the same neural types (Buonomano and Merzenich, 1998).

The way we conceive neural types is reminiscent of the contemporary mechanistic philosophy of neuroscience (see Craver and Kaplan, 2011). The mechanists hold that a mechanism produces the phenomenon of interest to the neuroscientists. However, we would prefer to put this in terms of the theory of identity because the philosophical mechanism, when causally interpreted, faces the issue of dualism (see Against Causal Accounts of NCCs). Thus the phenomena of interest, i.e., states of phenomenal consciousness, are not causally produced by the mechanism; they *are* this mechanism. Various phenomenal types are to be straightforwardly identified with various neural type-mechanisms.

### Putting It Together

What is the relationship between the two type-taxonomies, phenomenal and neural? Should we type the neurophysiological processes according to phenomenal criteria, or vice versa? Should they be typed entirely independently? We doubt that the latter is an option. On the one hand, phenomenal and neurophysiological states are typed by sets of very different characteristics – by phenomenal properties on the mental side, by neuroscientifically salient properties on the brain side. This could lead one to believe that to prefer the independent classification of both domains is the way to produce the right types. On the other hand, producing both taxonomies in complete isolation from one another and hoping that one day they will match perfectly is a dubious project. Such a meeting of types could never happen. We need a bridging procedure that brings phenomenal and neural types together.

This bridging procedure includes systematic NCC measurement in various sensory modalities. Without it, neuroscientific typing will produce taxonomies that will take into account only neuroanatomical (such as cytoarchitectonic) properties, whereas mental typing will produce taxonomies that will in no obvious way correspond to these neuroanatomical maps. (Think of the early introspectionists and their maps of “atoms of experience” – Revonsuo, 2010, pp. 52–53; Schwitzgebel, 2011, chapter 5). In short, typing mind and brain events and putting them together in a one-to-one correspondence relation is a jointly coordinated process based on the practice of searching for NCCs. We have already seen this in the previous subsection in which we defined neural types according to the neural mechanisms producing types of phenomenal states. These neural mechanisms cannot be uncovered in isolation from the phenomenal types. We agree with Viola (2017, pp. 164–165): one should prefer such type-taxonomies as enable systematic mapping between the entities in both domains, the phenomenal and the physical.^11^ This is not just pragmatically motivated advice. It is a *conditio sine qua non* of empirical consciousness research.^12^

**FIGURE 5:**
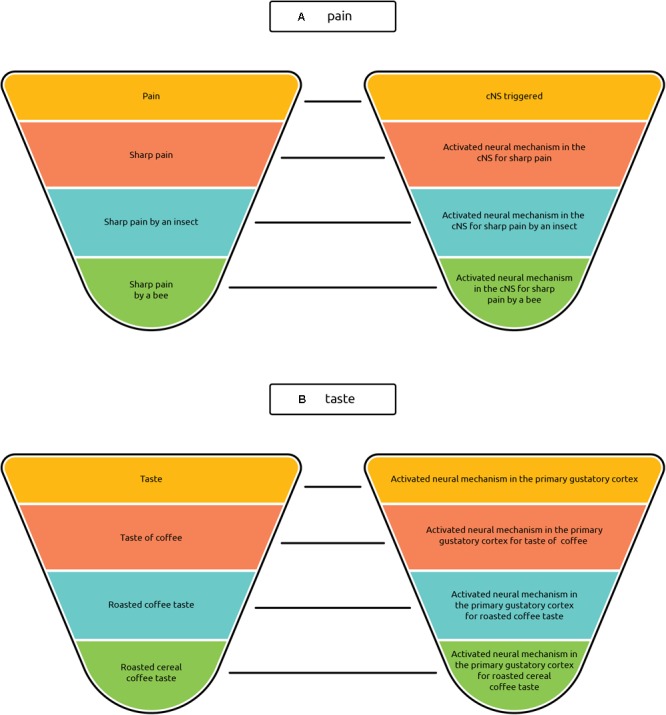
Simplified hierarchies of types. The diagrams show a one-to-one correspondence between simplified hierarchies of types. **(A)** displays the simplified hierarchy for phenomenal types of pain, **(B)** for phenomenal types of taste. On the right halves of both pictures, the corresponding neural types are assorted. Both the phenomenal and neural types are ordered according to the degree of generality: on the top the more general types are located, at the bottom the least general. An activated central nociceptive system (cNS in the diagram) is considered the neural correlate of pain; activation of the primary gustatory cortex is considered the neural correlate of taste (Chen et al., 2011; Gazzaniga et al., 2014, p. 176; Peng et al., 2015).

The so-called Heuristic theory of identity (HIT) proposed by Bechtel and McCauley consists in the following claim: “Scientists often propose identities during the early stages of their inquiries. These hypothetical identities are not the conclusions of scientific research but the premises” (Bechtel and McCauley, 1999, p. 69). These postulated type-identities are one of the sources of empirical progress in neuroscience; during research, they are further tested and refined. Now, some authors believe that postulating mind-brain identities is a methodologically dubious or perhaps even harmful step. For instance, Gamez (2014, p. 8) holds that “[w]hile it is possible that some version of identity theory or physicalism is correct, it would be controversial to base the scientific study of consciousness on this assumption, which would undermine our ability to gather data about the correlates of consciousness in a theory-neutral way.” In a similar vein, Paulewicz and Wierzchoń (2015, p. 238) claim that the empirical research of consciousness should proceed without introducing “unnecessary, untested and possibly confusing assumptions,” the identity theory presumably being one of them. In our view, these criticisms are misguided. Postulating mind-brain identities cannot affect empirical research into the neural correlates of consciousness in any negative way – indeed, quite the reverse. Besides the heuristic advantage of the prior assumptions of mind-brain identities that Bechtel and McCauley mention, adopting identity as a vertical mind-brain relation ensures that the coordination of phenomenal and neural processes is as tight as might be. As we will see in the next section, all other non-causal vertical notions construe a looser relation between phenomenal states and their neural substrate. This might please the anti-reductivists, but it puts in question the ontological status of the phenomenal and can potentially undermine the value of neuroscientific research for understanding consciousness: if the states of phenomenal consciousness are only loosely connected to their neural substrate, does scrutinizing the properties of this substrate really advance our understanding of how consciousness arises in nature?

The ultimate goal of the NCC research is to find the mechanism of consciousness itself (what Fink, 2016, calls *general* NCC). This search goes beyond the pairing of phenomenal and neural types. It simply is a search for the common neural mechanism of *all* states of consciousness, no matter how we type them (and their neurophysiological counterparts). This content non-specific neural activity, be it neural recurrence (Lamme, 2015), thalamocortical reentry loops into the dynamical core (Tononi and Edelman, 1998), microactivations of essential nodes distributed in the cortex (Zeki, 2003), or any other non-specific neural mechanism, is of such a nature that whenever it is activated, states of phenomenal consciousness are also present.^13^ It seems to be clear, though, that we can only reach this end-point of inquiry by working first with correlated phenomenal and neural type-taxonomies, gradually isolating the common neural mechanisms that they all share.

## A Plea for Identity Theory in Consciousness Research

The science of consciousness aims to elucidate the relationship between mental and brain phenomena. It cannot do this without a great deal of empirical work, but this work in itself is not enough. We need to *interpret* what the scientists are doing and what they are measuring in their labs. Consciousness researchers cannot dispense with this more theoretical aspect of their project. They cannot really afford to remain “vertically neutral.” Sooner or later they will need to move from the concept of the neural correlates of conscious states to some more robust notion accounting for the mind-brain relation.

The theory of identity fits the bill nicely. It is a simple and elegant metaphysical explanation of the mind-brain connection. Why do the neural states and states of consciousness correlate? Because they are *one and the same thing*. Claiming the identity of phenomenal and neural states enables one to describe their nature simultaneously. Examples from the history of science show that identifying two phenomena which were previously thought to be distinct brings rapid advances in the understanding of both. For instance, the discovery of the identity of lightning and electrostatic discharge enabled the use of all the knowledge amassed independently for both these phenomena in synthesizing a single explanatory account of their nature and behavior. Identifying brain processes with states of phenomenal consciousness could bring similar advances.

The theory of identity can be taken to be a form of *inference to the best explanation* – as some of its advocates, including Place, suggest. We cannot take the systematic mind-brain correlations to be a direct *proof* of the theory of identity, but we can hold that the theory of identity best explains them. The theory of identity, though, is not the only non-causal vertical metaphysical relation between the states of consciousness and brain processes. The literature distinguishes a number of such relations; each of them can claim to be the best explanation of regular mind-brain correlations. When compared to the notion of identity, these alternative materialist accounts work with the less reductive notions such as *constitution, multiple realization* and *supervenience*. These are all broadly naturalist: they all want to find a place for the phenomenal mind within and not beyond the physical world. Bennett (2011) puts them all in the general category of “building relations.” She shows that they are conceptually intertwined and also that there is a lot of room for disagreement as to the cogent characterization of any of these notions. We do not believe any of these relations to be superior to identity as a vertical mind-body relation. However, there is no space here to consider all these relations in detail. We shall only briefly indicate why we prefer the relation of identity over these less reductive notions.

*Constitution* is a one–one relation between distinct entities. It is not really appropriate for characterizing mind-body relations. One can apply it only to co-located, distinct objects such as a statue and a lump of clay. The lump constitutes the statue without being identical to it: transformations of its shape will cause the statue to disappear, while the lump continues to be the lump of clay it is. This cannot be the case in mind-brain relations. Transformations in neural patterns will either change the identity of both the neural state and the phenomenal state, or, if the transformations are radical enough, they will erase either the mental state or both the mental and the neural state.

The more frequently employed alternative to identity is that of *multiple realization* (see Aizawa and Gillett, 2009). The theory of multiple realization claims that the same phenomenal state can be realized in different substrates – not just in the human brain but also in the brains of some animals and even in artificial systems such as robots. A dependence upon types is clearly no less present in multiple realization theory than in the theory of identity. Multiple realization is a *type-multiple type* relation, i.e., it allows one-to-many mappings between phenomenal and physical types.^14^ It is not a theory of token-token relations. Multiple realization can be formulated as a relation holding (1) across different species, but can be also formulated as a relation (2) within one species, such as the human species, or even (3) within one individual (i.e., intrapersonal multiple realization). Thus, phenomenal type P is realized in organism H by neural type G, but by a different neural type, K, in a different organism, W (or in the same organism at a different time).

We accept that neural substrates realizing the same phenomenal type in different organisms can differ, within certain bounds. However, this is just a thesis of variability within a type, not a multiple realization. Variability is everywhere in nature but that does not imply that everywhere in nature there is also a variability of the realizing types (cf. Polger and Shapiro, 2016, p. 67). Multiple realization requires that the same type of mental state is realized by relevantly different neural, or more generally material realizers. However, as Polger and Shapiro (2016) show with a number of examples, many cases of phenomena that appear to be multiply realized are in fact implemented by realizers that are not relevantly different. Among those that, according to them, do not meet the relevantly difference criterion belong such cases as the neural plasticity of somatosensory cortex for processing tactile information of monkey’s hand, implementation of memory function, and jamming avoidance response in different electric fish. Moreover, they complement their analysis by pointing out that in other cases, the material substrate of a psychological state does change, but so does the state itself. For instance, one of the seemingly strong cases of multiple realization are the rewired brains of a group of ferrets (von Melchner et al., 2000). In the experiment, the ferrets’ retinal axons were rerouted in such a way that their auditory cortices started receiving the visual information. Soon, the ferrets began to adapt to the new situation. However, as Polger and Shapiro (2016, p. 95) note, the rewired ferrets’ discriminatory abilities significantly degraded. Multiple realization requires that the discriminatory performance and therefore the perceptual phenomenal states driving it remain unchanged. The rewired ferrets therefore cannot constitute an example of multiple realization.

In sum, unless the differences in neural realization are radical enough, they are to be conceived as intra-type variations which do not constitute multiple realization. The neural substrate of seeing blue can somewhat differ in different organisms, or even within a single organism at different times, but it will be the same neural type nonetheless. In this respect, we are in agreement with Polger and Shapiro when we claim that the differences within a neural type providing the substrate for a type of mental state constitute variability, not multiple realizability. As we indicated in Section “The Science of Consciousness Needs a Notion of Type,” this variability may be higher within the more general types in the hierarchy of types. There is thus no need to resort to multiple realization in order to account for ordinary natural variability. If, on the other hand, the differences in material implementation become too extensive, it becomes very likely that the mental type will not remain the same. This is the moral of the ferret example.

The identity theory sensitive to various levels of generality of types thus covers a number of cases of apparent multiple realization. This should allay the fears of many philosophers that the theory of identity is overly restrictive. However, the type theory of identity does rule out more substantial divergencies in the material substrate of mental states, especially across different biological species or non-living artifacts. But these, we believe, are not very plausible, given the available evidence. As has been noted by some authors (notably by Bechtel and Mundale, 1999, p. 203; Polger, 2004, p. 13, p. 30; Prinz, 2012, p. 275), it is difficult to ascertain that *the same* type of mental state is realized in a wildly different material substrate. For instance, the fact that other creatures have different brain architectures might be more naturally taken to imply that their conscious phenomenal states *differ*, not that they are the same as in us, humans. And the fact that artificial systems do not have brains at all might be further taken to imply that they cannot phenomenally experience anything. Even if other creatures or artifacts react in relevantly similar ways to similar stimuli as we do, this is not sufficient to rule out that they have different phenomenal states than we do – or no phenomenal states at all.

Another shortcoming of multiple realization as a vertical mind-body relation is that it can hold at most for the very general types within the type-hierarchy. This is also clear from the examples usually given of allegedly multiply realized phenomenal states. For instance, Putnam (1967) puts forward a claim that *pain* can be implemented not just by a human brain but also by the nervous system of an octopus, although the physical types realizing this phenomenal type will significantly differ in those two creatures. However, the further down we descend the hierarchy of phenomenal types, the more unlikely it is to see those phenomenal types as capable of being multiply realized. We find it implausible that types such as “a taste of Mandurian Primitivo” could be multiply realized. Identity, on the other hand, covers all instances of all phenomenal types without a single exception.

Finally, let us consider *supervenience*. Supervenience shares with identity the demand that any difference in phenomenal states, no matter how insignificant, must correspond to changes between the neural states implementing them. That is, phenomenal states supervene on neurophysiological states iff any change in a phenomenal state is conditional upon a corresponding change in the neurophysiological state (Kim, 1998). As with realization, supervenience is a weaker vertical notion than identity: supervenient states and processes are ontologically dependent on their subvenient (neurophysiological) basis, but they cannot be reduced to them. As far as we can tell, this is the only difference between supervenience and identity. However, if this is the case, we see no reason to prefer supervenience over identity, except anti-reductionist scruples. We do not share those scruples. On the contrary, we believe that succumbing to these scruples only makes matters worse for the consciousness science community. Whereas the identity theorist firmly accommodates all phenomenal properties in the material world, the supervenience theorist leaves their ontological status unexplained. His ontological business is, therefore, far from finished.

In short, it seems to us that identity has the most advantages as a vertical mind-body relation. Some authors go even further. Orly Shenker (unpublished) takes the radical stance that the only two genuine ontological relations that obtain in nature are those of identity and causation. This picture of things is quite simple. Since vertical mind-body relations cannot be causal (as per the argument in Section “Against Causal Accounts of NCCs”), they have to be the relations of identity. Our intention, though, is not to completely exclude other possible mind-brain relations. We do not have a knock-down argument against any of these alternative materialist notions. It might be that realization, supervenience or some other relation can serve as well or even better than identity.^15^ However, we know of no advantages that such notions have over identity. It seems to us that the only real virtues these notions have are those that they share with identity. Thus, both multiple realization and supervenience mimic the theory of identity in anchoring phenomenal properties in the material world. They both want to be seen as claiming that the phenomenal in some sense is the material, yet they also balk at reduction and want to create a special niche for phenomenal properties – that is, they want to picture them as a bit “more” than material. However, what constitutes this “more” is never made clear.

## Conclusion

The psychologist Ullin T. Place, founder of the modern brain-mind identity theory, saw the theory as a means of overcoming the philosophical obstacles of the empirical study of consciousness (Place, 1988, p. 211). By observing the current research, one might come to conclusion that these obstacles are a thing of the past: consciousness research is blooming and empirical researchers do not bother much with philosophical conundrums about mind-body relations. However, perhaps they should care about them rather more. Overgaard (2017), recounting the challenges of future consciousness research, points out that one of these challenges will be to reintegrate the metaphysical issue of the mind-body problem into the scientific work, because arguably the whole of consciousness research *is* about the mind-body problem, i.e., about how exactly it happens that the states of the brain carry the subjective states of phenomenal consciousness. As Overgaard points out, and as we stressed in the preceding section, “it seems not to be the case that evidence that perceptual experience is associated with—say—activity in primary visual cortex also provides evidence to determine whether consciousness should be seen as—say—identical to or metaphysically different from brain activity” (Overgaard, 2017, p. 3). We will need, Overgaard adds, something “extra” to cope with this challenge.

One intriguing possibility he mentions is that experiments will be designed to test theories put forward in the framework of the philosophy of mind. Unfortunately, no-one knows how to do this, or even whether something like this is possible. The second, less ambitious alternative Overgaard mentions is that experimental consciousness researchers could work more closely with theoretical consciousness research. We see our contribution in this paper as a step in this direction. We claimed above that without specifying the nature of the mind-brain connection, consciousness research will never be complete, our thesis being that the neuroscientists of consciousness should not work with causal models of brain-mind relations, and that they should replace them with the non-causal model of the identity theory. We have also indicated how the theory of identity fits the purposes of NCC research, which lies at the heart of the empirical science of consciousness, and why it can be taken to be superior to other notions as a “vertical” mind-body relation.

Apart from that, we also believe that by accepting identity, neuroscientists could bring clarity into their theoretical commitments. For instance, when Frith et al. (1999) try to characterize the relationship between brain states and phenomenal experiences, they use the terms “association” and “correspondence” (on pp. 105 and 107), yet this is hardly more informative than the metaphysically empty notion of correlation. Neither of the terms seem to cast any new light upon the mind-body problem. The notion of identity is much more informative and may provide a meaningful metaphysical framework for neuroscientific hypotheses about consciousness. By focusing on some aspects of NCC research we also discovered that the theory needs to be clear about the notion of *type*. Therefore, in Section “The Science of Consciousness Needs a Notion of Type,” we proposed a systematic hierarchy of phenomenal and neural types. We anticipate that this hierarchy might help scientists achieve greater clarity regarding the type-level at which they are working, on both the phenomenal and neural sides. This, in turn, could lead to more precise theories of the neural correlates of consciousness.

We have talked much about correlation in this paper. However, if the phenomenal states of mind are *identical* to those states of the brain with which they systematically correlate, the language of “correlation” ultimately becomes obsolete. Both states are not just correlated, they are the same. If the theory of identity is widely accepted, a new set of terms should be devised to clarify the nature of the tight connection between phenomenal and brain states.

## Author Contributions

Both authors listed have made a substantial, direct and intellectual contribution to the work, and approved it for publication.

## Conflict of Interest Statement

The authors declare that the research was conducted in the absence of any commercial or financial relationships that could be construed as a potential conflict of interest.

## Footnotes

1 We follow Schwitzgebel (2016) in defining states of phenomenal consciousness in a very non-committal, “innocent” way. These states are picked out by their phenomenal features, but no commitment to any theoretical postulates about their nature (such as whether they are non-relational, representational, incorrigible, etc.) is made.

2 The mapping Fink has in mind is a *bijection*, a one-to-one mapping between mental and physical types. Other kinds of mappings – one-to-many and many-to-one – are possible, but these are not the mappings admissible under the type theory of identity which requires a one-to-one correspondence between mental and physical types.

3 It should be emphasized that we have in mind the content-specific neural correlates of conscious states; there are also neural correlates of the so-called *background states of consciousness* such as being awake, being in a dreamless sleep, or being in a coma. In this paper we shall ignore the neural correlates of the background states of consciousness.

4 In the same vein, what happens at the retina and in the optic nerve is the CNCC of visual perceptions, etc.

5 N_2_, as a complex neural event, is constituted of substantial causal electrophysiological and chemical processes. Thus if the claim that phenomenal conscious state P is identical with N_2_ is true, P might be seen as involving causal interactions within N_2_. This sort of causality, though, does not clash with our formulation of the identity of P and N_2_; it is not causality in the vertical sense.

6 Searle (1992, Ch. 4–5) and Stephan (1999).

7 Note, though, that even this weaker theory of identity does not deny the existence of mental and neural types. It only denies the systematic correspondence between the two. It should also be noted that Davidson and Fodor claim token identity only for propositional attitudes and there is, thus, no token identity theory for states of phenomenal consciousness; see Kim (2012).

8 https://www.iasp-pain.org/terminology?navItemNumber=576

9 We hope that what we say about neural types could, if necessary, be adapted to alternative approaches to NCCs such as molecular-cellular accounts (Bickle, 2007; Prentner, 2017) or topological accounts which locate the NCCs in the strength of connectivity between various brain centers and areas (Kostic, 2014).

10 This is what Koch et al. (2016) call the “HOT zone.” In contrast to other dominant theories in the NCC research, the HOT zone theory excludes frontal areas from playing a role in determining phenomenal consciousness.

11 For one such generalized attempt see **Figure 5**.

12 Although both taxonomies are to be tightly coordinated, we believe that the phenomenal type-taxonomy is primary. It guides the whole process. We begin with salient mental types, at least with some crude basic categories high up in our hierarchy of mental types, and start correlating their incidence with occurrences of particular brain-states, which are far less differentiated amongst themselves.

13 In Marvan and Polák (2017) we propose a dual theory of phenomenal consciousness. This theory allows one to distinguish the general NCC mechanism from the content-specific mechanisms producing the particular phenomenal qualities of the mental states. The dominant, unitary conception of phenomenal consciousness does not permit this strategy.

14 Or between phenomenal and physical *properties* – as Figdor (2010, p. 419 n. 1) noted, “type” and “property” are often used interchangeably in the multiple realization literature.

15 At the end of Section “Introduction,” we defined the NCC in such a way that it does not include necessary conditions, only sufficient. Note that as a consequence of this weakening of Fink’s definition of the NCC, it could be used even by theorists that do not subscribe to the theory of identity. Notably, it could be used by defenders of multiple realizability. However, if the theory of identity proves to be the correct metaphysical solution to the mind-body problem, one can always tighten the conditions within the definition of the NCC by adding Fink’s “natural necessity” clause back.
